# Approaches to the discovery of non-invasive urinary biomarkers of prostate cancer

**DOI:** 10.18632/oncotarget.25946

**Published:** 2018-08-21

**Authors:** Andrej Jedinak, Kevin R. Loughlin, Marsha A. Moses

**Affiliations:** ^1^ Vascular Biology Program and Department of Surgery, Boston Children’s Hospital, Boston, MA, USA; ^2^ Department of Surgery, Harvard Medical School, Boston, MA, USA; ^3^ Department of Urology, Brigham and Women’s Hospital, Boston, MA, USA

**Keywords:** prostate cancer, biomarkers, non-invasive, proteomics

## Abstract

Prostate cancer (PCa) continues to be one of the most common cancers in men worldwide. Prostate specific antigen (PSA) measured in blood has been used for decades as an aid for physicians to detect the presence of prostate cancer. However, the PSA test has limited sensitivity and specificity, leading to unnecessary biopsies, overdiagnosis and overtreatment of patients. For these reasons, there is an urgent need for more accurate PCa biomarkers that can detect PCa with high sensitivity and specificity. Urine is a unique source of potential protein biomarkers that can be measured in a non-invasive way. This review comprehensively summarizes state of the art approaches used in the discovery and validation of urinary biomarkers for PCa. Numerous strategies are currently being used in the discovery of urinary biomarkers for prostate cancer including gel-based separation techniques, mass spectrometry, activity-based proteomic assays and software approaches. Antibody-based approaches remain preferred method for validation of candidate biomarkers with rapidly advancing multiplex immunoassays and MS-based targeted approaches. In the last decade, there has been a dramatic acceleration in the development of new techniques and approaches in the discovery of protein biomarkers for prostate cancer including computational, statistical and data mining methods. Many urinary-based protein biomarkers have been identified and have shown significant promise in initial studies. Examples of these potential biomarkers and the methods utilized in their discovery are also discussed in this review.

## INTRODUCTION

Prostate cancer (PCa) is the most prevalent cancer in males and it is estimated that approximately 116, 000 men living in USA were diagnosed with prostate cancer in 2017 [1]. The majority of the men (62%) diagnosed with PCa are 70 years or older with a median age of diagnosis at 66 years, thus reflecting the disease typically present in older men [2]. The majority of prostate tumors arise from the epithelial cells of the prostate peripheral zone with approximately 20-30% arising in the transition zone [3]. The most common non-malignant prostate disease significantly affecting aging men is benign prostatic hyperplasia (BPH), which is generally a disease of the transition zone [4]. These two prostatic diseases share similar symptoms and currently no reliable test exists with high specificity and selectivity that can differentiate between these two prostate diseases [5]. Another critical challenge is to differentiate between indolent or localized PCa from aggressive cancer [6] and reliably identify patients who would benefit from an active surveillance program [7]. Currently, a prostate cancer diagnosis is based on age, family history, race, prostatic digital rectal examination findings (DRE) and elevated levels of prostate specific antigen (PSA) in blood. However, none of these can reliably differentiate whether the patient has benign disease or prostate cancer. Therefore, a diagnostic dilemna exists and clinicians often recommend a prostate biopsy. This is a highly invasive approach, which is associated with high risk of bleeding, infection, urinary difficulty and hospitalization for signs of prostatitis or urosepsis, hematuria and hematospermia [8]. In addition, prostate biopsies can lead to sampling error, either missing the significant lesion or cancer completely [7], leading to false-negative results [9]. Thus, there is a pressing clinical need for an accurate PCa diagnosis to decrease unnecessary prostate biopsies [6].

In 1986, to guide clinical decision making, the US Food and Drug Administration approved the PSA test as a diagnostic, screening, and monitoring tool for the early detection of prostate cancer [10]. Prostate specific antigen (PSA) is a kallikrein serine protease (hK3) encoded by the KLK3 gene [11]. It was first purified in 1979 [12] and detected in serum in 1980 [13]. PSA is a 30-33 kDa protein [4], biologically responsible for semen liquefaction [14] that is secreted into the seminal fluid by luminal epithelial cells of the ducts and acini in the prostate. Normal basement membranes of prostatic ducts and acini as well as prostatic stroma limit the PSA release into the blood circulation [4]. Human kallikrein-3, also known as PSA, became the most widely used serum biomarker for the detection of PCa [6]. However, an elevated serum prostate-specific antigen (PSA) can be detected with either benign or malignant growth of the prostate [5]. PSA also can be elevated in prostatitis or physical trauma of the prostate thereby indicating pathologies of the prostate gland that are not necessarily cancer [14]. In addition, manipulations of the prostate (digital rectal examination [DRE], biopsy, catheterization, ejaculation) can also lead to elevated levels of PSA in blood [15]. Interestingly, PSA was also found to be expressed in the periurethral glands [16], normal breast tissue and various tumors [17]. Unfortunately then, PSA's main drawback is lack of specificity and sensitivity leading to unnecessary biopsies, over-diagnosis and overtreatment of insignificant PCa tumors [11]. A negative prostate biopsy is found in 65% to 70% of men with a PSA between 4.0 and 10.0 ng/ml^-1^ and PSA has only a 25-40% positive predictive value to detect PCa [18, 19]. Up to 15% men with PCa have a PSA levels below 4.0 ng/ml^-1^ thereby leaving certain cancers undetected [20]. To address these issues, a variety of permutations of PSA (age-adjusted PSA ranges, PSA velocity, PSA density, and free PSA fraction) have been used to improve the diagnostic sensitivity and specificity of PSA screening [5]. Nevertheless, all these PSA variations have been unsatisfactory in their capacity to differentiate between BPH and PCa in a clinical setting [21]. The potential of misdiagnosis, harms and small benefits resulting from PSA screening [22] lead the United States Preventive Services Task Force (USPSTF) in May of 2012 to not recommend PSA screening for PCa [5]. To summarize, there is a critical need for better quality PCa biomarkers that are noninvasive, have improved accuracy, and improved risk stratification properties [6]. In addition, a unified approach fulfilling the Reporting Recommendations for Tumor Marker Prognostic Studies (REMARK) [23] criteria that includes prospective studies and identification of optimal combinations of biomarkers is needed and critical to accurately identify and validate reliable urinary protein biomarkers for PCa that would allow the clinician to make more accurate clinical decisions.

## URINE AS A SOURCE OF PROSTATE CANCER BIOMARKERS

Urine is a biofluid enriched with proteins that reflect the physiological or pathological state of major urological tissues, including the prostate [24]. Urine also contains proteins that are secreted or have come in contact with the prostate making it an attractive liquid biopsy source of prostate biomarkers. The human urinary proteome was reported to contain approximately 2,000 proteins, including membrane, extracellular and lysosomal proteins [25, 26]. However, a recent study reported a total of 6,085 proteins identified in healthy urine [27] representing approximately one third of the whole human proteome which is currently estimated to consist of approximately 20,000 proteins [28]. Approximately 150 mg of proteins [25, 29] and 1-4 g of peptides are excreted in human urine by a healthy person each day [29]. It has been reported that the total urine output is approximately 1.5 liter/day/person [26], thus providing a more than sufficient amount for proteomic analysis [30]. In contrast, only a few microliters are often available to collect from small animals such as mice [31] therefore urine volume might be a limiting factor for proteomic analysis such that samples might need to be pooled. Collection of urine is very simple, can be noninvasively [32] collected over time in large amounts, and readily archived for processing [33] without having a high proteome background such as plasma. Urine can be stored for several months at -20°C or for several years at -80°C without, in many cases significantly changing the human urinary proteome. It is known that the human urinary proteome changes with disease status, thus making urine one of the most attractive biofluids for discovery of prognostic, diagnostic and monitoring biomarkers. In summary, urine is one of the most interesting and useful biofluids in PCa biomarker discovery and can be obtained non-invasively in large quantities.

## PROTEOMICS IN PROSTATE CANCER BIOMARKER DISCOVERY

Proteomics is the large-scale study of proteins [33] which offers complementary information to genomic and transcriptomic studies essential for understanding complex biochemical processes [34]. It can also be defined as a postgenomic discipline that encompasses efforts to identify and quantify all the proteins of a proteome [35]. The proteome is the complete set of proteins found in living cells, tissues or organisms, representing the end result of gene transcription, translation and protein synthesis up through post-translational protein modification (PTM) [36]. However, analysis of the human proteome is very challenging due to its unique characteristics which include a high dynamic range of protein expression [37], alternative splicing events, interconnectivity of proteins into complexes and signaling networks [38] and significant complexity due to an excess of PTMs and sequence variations [37]. Protein activity, stability, localization, and function are also often modulated by PTMs [37, 39]. A number of human diseases, including cancer, have been previously linked to PTMs, such as protein acetylation, glycosylation, hydroxylation, and phosphorylation [37, 40]. Due to the complexity of the proteome, the progress of proteomics has been driven by the development of new technologies for peptide/protein separation, mass spectrometry analysis, isotope labeling for quantification, and bioinformatics data analysis. Mass spectrometry (MS) has emerged as a powerful technique to identify, characterize, and quantify proteins and their PTMs with high throughput and on a large scale [34]. MS is frequently used as a discovery tool with high sensitivity and specificity that plays a crucial role in biomarker discovery. In the past decade, MS methods have been employed in liquid biopsy approaches and the discovery of numerous protein biomarkers of various cancers thereby representing a cornerstone of protein-based cancer biomarker discovery [33].

### Proteomic strategies involving mass spectrometry

In mass spectrometry-based proteomics there are two distinct approaches in proteomics analysis: the less used, less mature “top-down” proteomics, and the almost universally used “bottom-up” proteomics [41]. Protein identification by top-down proteomics involves analysis of intact protein [39] without enzymatic treatment, followed by protein ionization and LC-MS analysis [28]. One of the advantages of the top-down approach is that it provides reduced sample complexity in comparison to the proteins digested and analyzed by using the bottom-up approach [37]. Another advantage is that it allows the characterization of proteoforms, protein isoforms and PTMs [42]. However, the top-down method has significant limitations compared to bottom-up proteomics due to difficulties with protein fractionation, protein ionization, and fragmentation in the gas phase [35]. In contrast, chemical or enzymatic treatment of proteins into smaller peptides, followed by MS analysis is characteristic of a bottom-up strategy [33]. Peptides are usually identified by specific bioinformatics tools to match tandem mass spectra (MS/MS) with the theoretical fragmentation patterns generated using a genomic database [34]. Also included in this category is the "shotgun" proteomics approach wherein the mixture of proteins in a sample is digested and then analyzed by mass spectrometry without first separating individual whole proteins [24]. An automated variant of shotgun proteomics named multidimensional protein identification technology (MudPIT) has been developed, which incorporates multidimensional high-pressure liquid chromatography (LC/LC), tandem mass spectrometry (MS/MS) and database-searching algorithms [43]. An important part of biomarker discovery is MS-based protein quantification where biomarkers are typically identified through changes in protein or peptide concentrations between sample groups [44]. Common methods rely on label-free quantification or label-based quantification of proteins or peptides [45]. Stable isotope label-based quantification can be categorized from a MS point of view as “isotopic” or “isobaric.” The main difference between these two methods is that isotopic approaches such as SILAC (stable isotope labeling by amino acids in cell culture), ICAT (isotope-coded affinity tag) and ICPL (isotope-coded protein label) methods measure ion intensities of light and heavy isotopes of a peptide for quantification at the MS level. In contrast, isobaric methods including TMT (tandem mass tags) and iTRAQ (isobaric tags for relative and absolute quantitation) quantify peptides at the MS/MS level based on comparison of the reporting peaks with different isotopic labeling [46]. Our group has recently conducted a study utilizing the iTRAQ approach to discover new urinary biomarkers that could distinguish between benign prostate hyperplasia (BPH) and localized prostate cancer. We utilized an 8-plex iTRAQ format in which four urine samples from patients diagnosed with BPH and 4 urine samples from patients diagnosed with PCa were analyzed. One advantage of using the 8-plex iTRAQ format is that it permits the simultaneous identification and quantification of multiple samples under the same experimental conditions. Moreover, this system also allows one sample to be used as an internal reference, thus allowing for cross-set comparison. We identified, with high confidence, 25 proteins whose levels were significantly different between these two groups. Surprisingly, these proteins range widely in function from cell assembly and organization, cell signaling, cell morphology, carbohydrate metabolism, cellular growth and proliferation, lipid metabolism, androgen and estrogen metabolism, DNA replication, recombination and repair, among others. Three proteins, β2 M (β2-microglobulin), PGA3 (pepsinogen 3), and MUC3 (mucin 3), were found to be significantly different between urine samples from BPH patients and samples from prostate cancer patients through univariate analysis. These proteins, either alone or when multiplexed, showed significant sensitivity and specificity in discriminating between patients with BPH and those with localized prostate cancer. In addition, these new biomarkers significantly increased predictive accuracy based on PSA categories from 0.734 to 0.812 when combined (P=0.004, Delong test for comparing ROC curves) [5]. In another study, we used tandem MS/MS mass spectrometry in combination with chromatography and zymography to identify high molecular weight gelatinase (HMW) species in urine from patients with different cancers. Distinct MMP fingerprints were identified for organ-confined prostate cancer vs. bladder cancer. MMP-9 dimer and MMP-9 monomer were multivariable predictors for distinguishing between patients with prostate and bladder cancer (*P< 0.001* for each) [47].

The label-free protein quantification approach is based either on the comparison of extracted peptide MS peak intensities from different biological samples (intensity-based quantitation) or on the total number of MS/MS acquired for the same peptide (spectral counting approach) to represent the relative abundance of this peptide in the mixture [34]. The sample processing is simple, relatively inexpensive (no labeling reagent involved) and offers greater dynamic range and proteome coverage compared to label-based methods [48]. In addition, this is a very high throughput technique frequently used in the discovery of urinary proteome biomarkers from human samples [49]. However, label-free approaches also have some disadvantages, such as reproducibility between sample runs [44], redundancy in peak detection, lower accuracy, a semi-quantitative nature, and the lack of unsuitability for low abundance and small proteins [49]. Contrary to quantitative protein profiling, which is an unbiased proteomic approach, targeted quantitative proteomics is a candidate-based method that permits specific detection of selected analytes in a complex system [46]. Targeted quantitative proteomics is based on a hypothesis-driven selection of the proteins of research interest, while nontargeted peptides are not analyzed [50]. Targeted detection and quantification of candidate biomarkers is generally achieved by selected reaction monitoring (SRM), also referred to as multiple reaction monitoring (MRM) [44]. The SRM/MRM technique is considered the gold standard proteomic quantification method for predefined sets of proteins [50]. SRM is a nonscanning method that is performed on triple quadrupole-type instruments (QqQ) [51]. The first quadrupole (Q1) is used to isolate specified precursor ions, the second quadrupole (Q2) serves as the collision cell to activate and dissociate the precursor ion, and the third quadrupole (Q3) is used to isolate the specific product ion [52]. This SRM technique offers high-throughput performances, high selectivity and sensitivity, wide dynamic range of measurements and high reproducibility [50, 53]. Nevertheless, the selectivity of this mass spectrometry is reduced by the resolving power of its mass analyzers such that interferences may require reanalyzing the samples. Despite the efforts undertaken to limit the shortcomings, the process still remains laborious and time consuming [54]. Recently, targeted mass spectrometry-based approaches have been widely used for quantitative proteomics studies and have been applied on to high resolution/accurate mass (HRAM) instruments, such as the quadrupole-time of flight (Q-TOF) and quadrupole-orbitrap (Q-OT) resulting in a substantial performance enhancement [55]. More precisely, analyses executed on quadrupole-orbitrap mass spectrometers operated in parallel reaction monitoring (PRM) mode leverage intrinsic high resolving power and trapping capabilities [53] thereby offering a clear advantage over the conventional SRM measurements executed on triple quadrupole instruments [55]. The potential of the PRM technique offers very high degrees of selectivity and analytical sensitivity, usually required to analyze peptides in complex samples, such as those used in biomedical research or clinical studies [53].

To accurately and quantitatively analyze data in MS-based approaches, computational methods and common search engines are used to help facilitate quick data analysis [33]. The most widely used computational methods are based on the use of protein sequence databases, search engines, de novo sequencing and spectral libraries [56]. Consequently, several well established applications for peptide identification are now being used worldwide including Sequest [57], X!Tandem [58], Mascot [59], MyriMatch [60] or Andromeda [61]. These tools first perform an in silico digestion of all proteins in a reference protein database to compute all candidate peptide sequences and then build a theoretical spectrum for each candidate peptide sequence [62]. The expected endpoint for most datasets obtained for research purposes is storage in public data repositories thus allowing access for other researchers [63]. Several well-known proteomics databases have been developed including, Global Proteome Machine Database (GPMDB) [64], PeptideAtlas [65], and the PRIDE database [66]. In addition, there are currently a few databases that are more specific to the human urine proteome such as MAPU [67], Sys-BodyFluid [68], HKUPP [69], Urinary Exosome Protein Database [70], Urinary Protein Biomarker Database [71], Mosaiques Diagnostics database [72] and UPdb [73].

### Proteomic approaches

**Figure 1:**
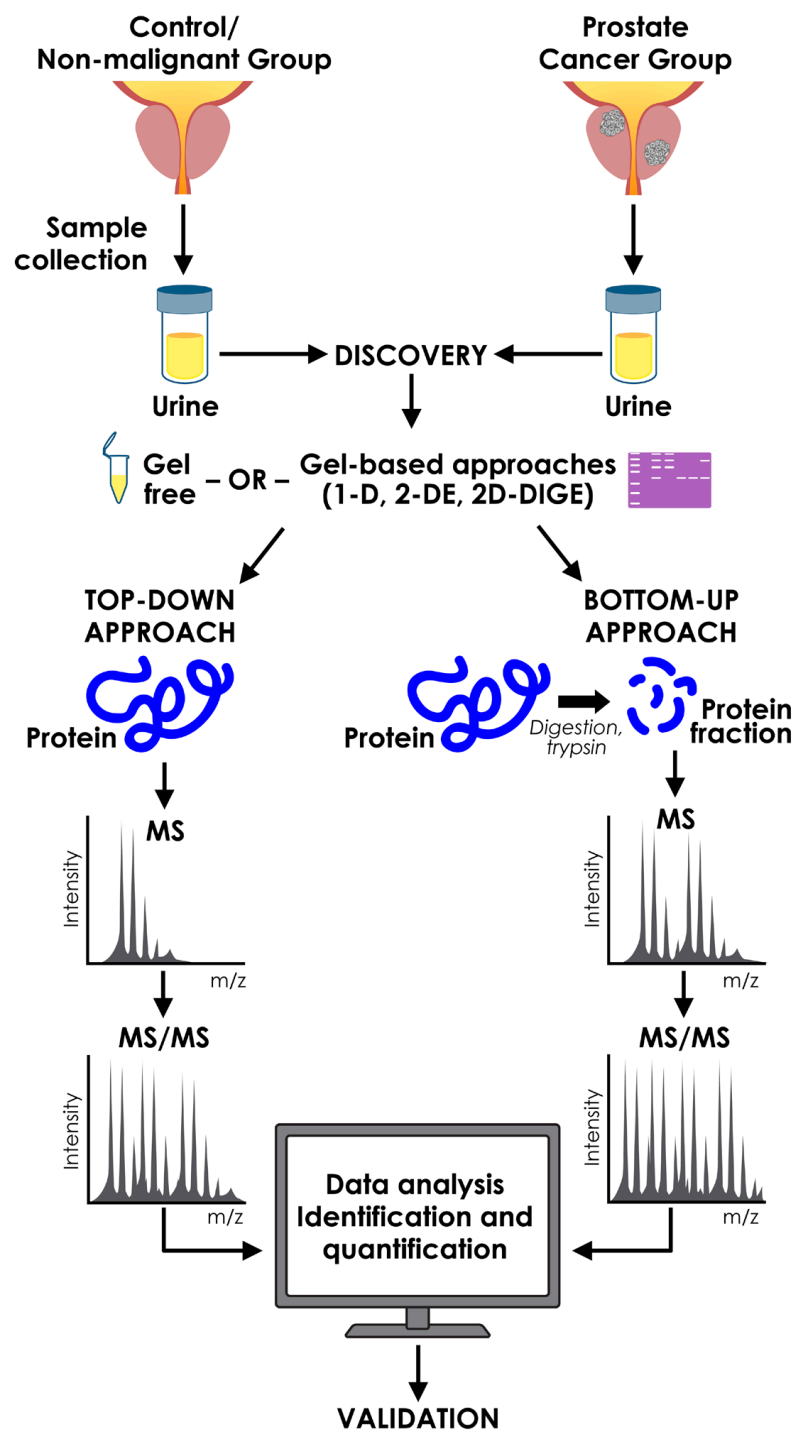
Proteomic approaches used in discovery of urinary prostate cancer biomarkers

In general, there are two commonly used proteomic separation techniques, gel-based and gel-free methods (Figure 1).

#### Techniques utilizing gels

The two most common gel-utilizing methods to fragment and set apart proteins in a gel-based substance are 1-dimensional (1D) and 2-dimensional (2D) gel electrophoresis. After gel electrophoresis, proteins are cut out from the gel, processed with a protease such as endopeptidase trypsin, and the resulting peptide fragments are examined using MS [33, 74]. In one of our studies, 1D SDS-PAGE gel electrophoresis in combination with column chromatography, zymography, and tandem MS (MS/MS) facilitated the identification of MMP-2 (*P* < 0.001) and MMP-9/NGAL (*P* = 0.003) by multivariable regression as independently predictive in differentiating prostate cancer patients and controls. In addition, MMP-9 and MMP-9 dimer were identified as multivariable predictors for differentiating prostate from bladder cancer (*P* < 0.001) [47]. In a recent study, 2-D DIGE gel electrophoresis coupled with MS and bioinformatics analysis identified serotransferrin (TF), alpha-1-microglobulin/bikunin precursor (AMPB) and haptoglobin (α-chain) (HP) as new urinary biomarkers, which could distinguish between BPH and prostate cancer. The area under the curve (AUC) for the individual proteins ranged from 0.723 for HP (p = 0.008), 0.738 for AMBP (p = 0.005) and 0.754 for TF (p = 0.002). In another recent study, 2-D PAGE followed by matrix-assisted laser desorption ionization-time of flight-mass spectroscopy (MALDI-TOF-MS) identified calgranulin/MRP-14 as a potential biomarker in voided urine that distinguishes between BPH and PCa [75]. Due to such drawbacks as a limited protein dynamic range and issues with incomplete digestion, gel-free proteomics soon became a preferred approach [7, 36].

### Comparison of acquisition methods, cost-effectiveness and reproducibility

Development of new technologies and tools in the last decade significantly accelerated progress in proteomics approaches and biomarker discovery. However, an open question remaining is the determination of which specific proteomic approach is the most appropriate to use in biomarker discovery and validation when using clinical samples [76]. Several studies have compared proteomic approaches for protein identification and quantification in human specimens. For example, 2D DIGE, ICAT and iTRAQ were compared in a protein profiling study [77] that showed that iTRAQ offered better quantitative reproducibility and higher sensitivity than 2D DIGE or ICAT [77]. In a separate study, iTRAQ 4-plex versus 8-plex were compared with respect to protein quantitation in human plasma samples. The data revealed that iTRAQ 8-plex provided more consistent ratios than the 4-plex without compromising protein identification and offered higher sample throughput than 4-plex [78]. Moreover, multiple studies compared label-free proteomics with label-based methods such as SILAC [79], 8-plex iTRAQ [76] or the 6-plex TMT approach [80] for protein identification and quantification. Taken together, these studies suggested that label-free proteomic approaches provide better protein coverage and outperform the label-based methods. Generally, shotgun MS approaches appear to be the preferred choice used for discovery studies, while directed and targeted MS methods are commonly used in verification and validation studies [79]. However, more “head to head” and “side by side” studies comparing the suitability of particular proteomic methods in biomarker discovery and development are needed.

Overtreatment and overdiagnosis of PCa patients significantly raises the cost of health care. Therefore, a novel economical, non-invasive approach, which would limit the number of biopsies, could also lead to the reduced cost. For example, analysis of urine from PCa patients performed by capillary electrophoresis mass spectrometry (CE/MS) outperformed the biopsy approach as well as the PSA test. Moreover, a majority of cost savings were related to the significant reduction in the number of necessary biopsies by 49% [81]. Cost-effectiveness can also be achieved by more accurate tests. For instance, the use of the SelectMDx test after PSA ( >3 ng/ml) as a second diagnostic tool to inform the need for prostate biopsies led to savings of €128 and an increase of 0.025 quality-adjusted life years (QALY) per patient versus the standard of care strategy (PSA+DRE) [82]. Addition of the Prostate Health Index (PHI) testing for men with elevated serum PSA is another example where the number of negative biopsies can be significantly reduced, leading to better detection and improved cost-effectiveness [83].

There is a limited literature regarding reproducibility of the urinary proteomic profile [84] and therefore a significant need to address this issue. Discovery proteomic methods including chemical labeling and label-free approaches showed, in general, a high degree of protein and peptide detection reproducibility in urine [85, 86]. Targeted proteomic approaches such as SRM/MRM demonstrated high reproducibility of detected proteins in urine samples and results were comparable to those obtained by ELISA [87]. However, more studies comparing proteomic approaches are needed.

### Advantages and disadvantages of the use of the proteomics approaches

While advances such as new instruments, techniques and software have led to the evolution of novel proteomic approaches for identification and validation of protein biomarkers, there also exist a variety of challenges and limitations associated with this approach. For example, gel-based techniques are frequently used in discovery but are not applicable in clinical settings due their time consuming nature, lack of high-throughput capabilities, requirement for high antibody quality and need for continuous optimization. MS-based methods are very sensitive, specific and generate significant amounts of information and, as such, are now gradually being incorporated into clinical settings. However, there are also some drawbacks associated with MS-based methods such as the need for expensive hardware, specific software, expensive equipment maintenance fees, high cost of certain chemicals, low-throughput and the requirement of highly skilled personal. Functional assays are highly specific and provide activity readouts, but their use can be limited due the availability and cost of commercial antibodies or reporter tags. ELISA-based methods are simple, fast, have high-throughput, can be performed by trained technician, and can be highly sensitive and specific. This technique is frequently used in validation of proteomic discoveries and in clinical settings as well. However, detection of only a single antigen at a time is an important limitation which is being addressed by the development of a number of multiplex immunoassays.

### Strategies with functional proteomic technology

Proteases play a key role in normal physiological functions in the body. However, dysfunction in the biological control mechanisms of proteases may contribute to various diseases, including cancer and cancer metastasis [88]. Therefore, detection and quantification of proteases in body fluids, including urine, may be useful for cancer diagnosis and prognosis as well as to monitor therapeutic responses in patients [33]. In particular, expression and activity of gelatinases, MMP-2 (72 kDa) and MMP-9 (92 kDa) have been shown to be significantly up-regulated in a variety of cancers, including prostate cancer. A sensitive and inexpensive method for analysis of gelatinases (MMPs) is gelatin zymography, which allows simultaneous measurement of both active and latent forms of MMP-2 and MMP-9 in biological fluids, including urine [89]. Our group used gelatin zymography to analyze urine samples from patients with organ-confined prostate cancer and samples with organ-confined bladder cancer as well as samples from control subjects. We found that MMP-9 dimer and MMP-9 were independent predictors for distinguishing between patients with prostate and bladder cancer (P < 0.001 for each). This study indicates that a tumor-specific urinary MMP fingerprint may noninvasively facilitate identification of cancer presence and type [47]. In another study, we have successfully used gelatin zymography to noninvasively monitor therapeutic efficacy in prostate cancer. Urinary MMPs were analyzed at the patient’s first evaluation, during radiation treatment and after the radiation therapy. We found that urinary MMP levels were higher in patients with local-regional cancer compared with normal controls. Moreover, MMP levels were significantly higher (*P* <0.001) in patients with metastatic disease at presentation than in patients with local-regional disease or normal controls (*P* <0.001). The MMP levels were also significantly associated with 1-year progression-free survival as was the individual MMP-2 trend (*P* = 0.004 and 0.001, respectively) [90]. These results were similar to our previously published data which found an increased incidence of urinary MMPs in cancer patients, including prostate cancer [91]. In summary, these data suggest that MMPs analyzed by gelatin zymography may play an important role in monitoring disease progression after radiotherapy and may also predict patient survival after therapy [90]. Recently, new non-invasive FRET (Förster resonance energy transfer)-based ratio-metric detection of hyaluronidase as a biomarker for bladder and prostate cancer has been reported. This assay uses a fluorescent substrate (termed HA–FRET) labeled with fluorescein as a donor and rhodamine as an acceptor for detection of enzymatic activity in synthetic urine. Digestion of the HA-FRET probe is measured with different concentrations of hyaluronidase via fluorescence emission and the extent to which FRET is released is dependent on the concentration of hyaluronidase [92].

Activity-based protein profiling (ABPP) has recently developed as an unconventional approach that is complementary to gene expression analysis and represents a tool to assist in decoding the overflow of genomic information [93]. Selective visualization of the active forms of specific enzymes by chemical probes termed activity-based probes (ABPs) is the ultimate goal of ABPP [94]. In contrast to substrate-based probes, ABPs covalently label the active site of a specific enzyme [95]. In general, ABPs consist of a reactive group for the covalent binding to the enzyme, a linker (spacer) for modulating reactivity and specificity of the reactive group and a reporter tag for characterization and purification of modified enzymes [96]. Detection of reporter tags can be done by various analytical platforms, including mass spectrometry, SDS-PAGE, fluorescence microscopy, and *in vivo* imaging [95]. ABPs have been successfully developed for various proteases, including serine hydrolases, cysteine proteases, metallohydrolases, aspartyl proteases and the proteasome [97]. Recently, activity-based protein profiling-guided medicinal chemistry has been used for the discovery and development of the highly potent and selective inhibitor of serine hydrolase KIAA1363 in prostate cancer cell lines [98]. Moreover, activity-based protein profiling was also used to identify platelet activating factor acetylhydrolase 1B2 and 1B3 (PAFAH1B2 and PAFAH1B3) which were consistently upregulated by various human oncogenes. Pharmacological blockade of PAFAH1B2 and PAFAH1B3 impaired cancer pathogenicity across a number of different types of cancer cells, including prostate cancer [99]. Lastly, activity-based proteomics (ABPP) used for carcinoma enzyme activity profiles may be more clinically valuable than expression-based proteomics for certain cancers, including prostate cancer [100]. Therefore, the application of ABPP techniques offers a versatile tool in liquid biopsy approaches for protein biomarker discovery and the development of highly potent and selective inhibitors.

### Data analysis and bioinformatics

Bioinformatics plays a key role in the analysis of MS data [101] used to identify candidate biomarkers. Proteomic experiments often generate large datasets with massive amount of data [102] which need to be reduced into a short list of proteins. This represents challenge to accurately interpreting data and identifying candidate biomarkers. Therefore, functional annotation analysis of protein datasets through computational tools is critical for interpreting the results of high-throughput proteomics [102]. Pathway analysis may be applied to proteomic data to narrow down interesting biomarkers. Some of the most common tools used in pathway analysis are Ingenuity Pathway Analysis [103], KEGG [104] and MetaCORE [105]. We have also used Ingenuity Pathway Analysis (IPA) to determine differentially expressed pathways and functions in localized prostate cancer (PCa) as compared to BPH. IPA identified proteins in a number of different functional categories including cell assembly and organization, cell signaling, cell morphology, carbohydrate metabolism, cellular growth and proliferation, lipid metabolism, androgen and estrogen metabolism, and DNA replication, recombination and repair, among others. Additionally, network analysis identified differences in many focus hubs (e.g. NFκB, ERK1/2, Collagen, TGFβ, PI3K, and p38 MAPK) with a high degree of interactivity. Furthermore, gene ontology tools are very valuable for better understanding the potential biological function and molecular process of a given protein [102]. A number of online gene ontology tools perform these analyses including, DAVID [106], BiNGO [107] and AmiGO [108]. In addition, interaction networks tools are essential in the visualization and interpretation of biological processes [102]. For instance, STRING [109] and Cytoscape [110] are some of the widely used interaction network software. Literature or text mining is another valuable informatics approach that can be useful in biomarker discovery. Several text-mining tools are available such as iHOP [111], Chilibot [112] and Biotext search engine [113]. Moreover, public availability of proteomics data from existing data repositories greatly assist researchers as they interpret their proteomics data and can significantly contribute to the generation of new hypotheses [102]. Recently, the ProteomeXchange (PX) consortium containing several member databases, including PRIDE and PeptideAtlas has been developed to enable better integration of public repositories and the coordinated sharing of proteomics information, maximizing its benefit to the scientific community [114].

## VALIDATION OF CANDIDATE BIOMARKERS

The biomarker validation stage is a necessary requirement indispensable for successful biomarker implementation in clinical practice [115]. Only the most promising biomarkers that were previously verified should be considered for the evaluation. Moreover, the validation phase should include prospective and retrospective validation for general population screening [45]. Validation must be executed in an independent, appropriately large sample set that reflects the heterogeneity of the targeted population [116]. ELISA (enzyme-linked immunosorbent assay), immunoblot, protein arrays, immunohistochemistry (IHC), quantitative MS for protein and peptide analysis, and chemoproteomic assays are the most frequently used methods for protein biomarker validation [33]. However, only certain of these methods can be applied for validation of urinary biomarkers. Currently, due to its high throughput, high specificity, simplicity and high sensitivity for quantification of proteins, ELISA is considered to be the gold standard technique for validation of protein biomarkers [117] in biofluids, including urine. There remain several drawbacks regarding ELISA use in large validation studies including a limitation in the detection of a single antigen low-dynamic range, high cost of ELISA development and lack of specific antibodies [116]. Multiplex immunoassays have been developed to tackle some of the limitations of classical single-antigen based detection ELISA assays, including Assay platforms MULTI-ARRAY (Meso Scale Discovery), Bio-Plex (Bio-Rad Laboratories), A^2^ (Beckman Coulter), FAST Quant (Whatman Schleicher & Schuell BioScience), and FlowCytomix (Bender MedSystems) among others [118]. In particular, electrochemoluminiscence multiplex assay platforms offered by Meso Scale Discovery (MSD) are now being used for validation of non-invasive biomarkers [119] and clinically as well [120]. The MSD system consists of carbon electrode plates, which are pre-coated with captured antibodies against different targets at the bottom of each well. When the analyte, is applied it binds to the capture antibody followed by recognition of detection antibodies conjugated with electrochemiluminescent labels (SULFO-TAG) leading to light emission when the electricity is applied to the plates. MSD platforms come in 96- and 384-well formats, allowing quantification of 4, 7 or 10 analytes per well in a 96-well format and single or 4 analytes in 384-well format. This system permits high throughput and measurement of multiple targets with broad dynamic range, high sensitivity, low background, ease of use, high performance and compatibility with biofluids. The sample volume required is only 25ul/well for the 96-well and 10ul/well for the 384-well format, thus being suitable for analysis of small volume samples [121]. Recently, the multiplex proximity ligation assay (PLA) has gained increased clinical attention with respect to predicting severity of disease [122]. The PLA method is based on the ability of two or more antibodies to recognize targeted proteins by using DNA oligonucleotides bound to conjugated antibodies which can be then hybridized to form single DNA strands through ligation or polymerization [123]. Quantification is then performed by RT-PCR or by DNA sequencing. The 4-PLA (4 distinct antibodies) approach was used to investigate prostasomes (microvesicles from prostate cancer cells) as promising plasma biomarkers for PCa. The results revealed significantly elevated levels of prostasomes in blood of PCa patients compared to controls or patients diagnosed with benign disease. Moreover, the assay was able to differentiate patients with medium (7) or high Gleason score (8/9) from patients with low Gleason score (≤ 6), thus mirroring prostate cancer aggressiveness. This study suggests that the PLA method may represent a useful tool for assessment and prognosis of localized PCa [124]. Another commonly used method to validate urinary protein biomarkers is western blot (immunoblot) analysis. However, this approach can be used only on small cohorts due to its limitations regarding detection of single antigens, the requirement to optimize experimental conditions, labor intensity, lack of specific antibodies and lack of high-throughput capacity among other limitations. Nevertheless, these two conventional methods (ELISA and western blot), are often the first choice for validation of biomarkers [49]. Promising alternatives to the immunoassays discussed above are MS-based targeted approaches offering reduced costs, shortened lead-time, and greatly improved throughput [44]. Selected reaction monitoring (SRM) and multiple-reaction monitoring (MRM) are the two most common methods used for absolute quantification of proteins in combination with stable isotope dilution [49]. However, these methods also have some shortcomings that include limited commercial availability of isotopically labeled internal standards and high cost. The recent implementation of targeted high-resolution and accurate-mass analyses on fast sequencing mass spectrometers operated in parallel reaction monitoring (PRM) mode offers a clear improvement over the classical SRM/MRM measurements performed on triple quadrupole instruments [53, 55]. In addition, compared to SRM/MRM methods it provides improved selectivity, specificity, superior resolving power and better discrimination of the signal of the analytes from that of the matrix [53, 55]. In the future, the versatility of the MS-based targeted approaches may prove to be more cost-effective and less time-consuming than immuno-based approaches for urinary biomarker validation.

## NONINVASIVE URINE-BASED PROSTATE CANCER BIOMARKERS

Recent advances in proteomic technologies, molecular biology and better understanding of carcinogenesis with respect to PCa has led to the discovery of new protein biomarkers [11] and to innovations in liquid biopsy approaches. Protein-based biomarkers are frequently secreted into bodily fluids, including urine, and in contrast to DNA and RNA do not necessarily rely on the presence of cancer cells for detection [125]. In addition, nucleic acid-based markers are limited in their ability to integrate information associated with the downstream processing of proteins significantly affecting the information content of a biomarker [126]. The ideal biomarker should be highly sensitive, specific to PCa, not altered or expressed in other human tissues or diseases and the method of collection should be non-invasive. It is well accepted that every single biomarker has its own performance limits. Therefore, a panel of biomarkers, rather than single biomarker in isolation, could improve accuracy, decrease false positive values and better address the complexity of the disease. In addition, certain biomarkers may work better together than in isolation. Our longstanding interest has been to identify noninvasive diagnostic and prognostic biomarkers for variety of cancers, including prostate cancer [33]. The list of discovered urinary protein biomarkers including biomarkers discovered by us is summarized in Table 1. The summary exclusively focuses on proteins and does not include RNA, DNA, metabolomics, exosome or cancer cell-based biomarkers which are beyond the scope of this article. There are a number of novel emerging PCa biomarkers that should be noted. Recently, there has been an increase in interest of some tissue-based biomarkers for PCa including PTEN, ERG, FASN, MAGI-2, SPINK1 [127], CXCL12 [128] and glycoforms of PSA present in blood [129]. The alternatives to more traditional tissue-based or blood-based biomarkers are biomarkers present in human urine. The best known/studied emerging urinary biomarkers include aHGF, IGFBP3 [130] and OPN [130, 131], long non-coding RNA (lncRNA) biomarkers such as PCA-3, TMPRSS2, and PCa specific methylation biomarkers such as glutathione S-transferase P (GSTP1) [132]. In addition, circulating tumor cells (CTC), exosomes, cell-free DNA (cfDNA) and miRNAs [129] including miR-100/200b [133], miR-21 and miR-375 [134] have been reported and show promise as prostate cancer biomarkers. Currently, a few non-protein based urinary tests for prostate cancer are commercially available including PCA3 (Hologic, Marlborough, MA, USA), TMPRSS2:ERG (University of Michigan, MI, USA), ExoDx (Exosome Diagnostics Inc., Cambridge, MA, USA), SChLAP1 (GenomeDx Biosciences, San Diego, CA, USA) and SelectMDx (MDxHealth, Irvine, CA, USA) [6]. There are, to our knowledge, few if any protein-based non-invasive FDA approved and commercially available, reliable urinary tests for prostate cancer. Therefore, there is an unmet need and unique opportunity for the development of a non-invasive urinary protein-based test which can detect PCa with high sensitivity and specificity.

**Table 1:** Urinary biomarkers for prostate cancer

Urinary Biomarker	Method	Results	References
Transferrin	Immunoturbidimetric assay	Significantly increased levels in 18 out of 22 PCa patients	[137]
uTF	Chromogenic assay	Sen. 68%, Spe. 75%	[138]
MCM-5	Immunofluorometric assay	Higher levels of Mcm5 in their urine sediments than did men without malignancy (*P*<.001)	[139]
AMACR	Western blot	Sen.100%, Spe 58%	[140]
Calgranulin B/MRP-14	2D-PAGE, MALDI-TOF-MS	Present in four of six fluid samples from patients with cancer but in none of the fluid samples from patients with BPH	[75]
TB-15	ELISA	Sen. 41%, Spe. 92%	[141]
Uromodulin, semenogelin I isoform b preproprotein	MALDI-TOF	Sen. 71%, Spe. 67%	[142]
MMP species	Gelatin zymography, MS	Sen. 74%, Spe. 82%	[47]
12 protein panel	CE-MS	Sen. 89%, Spe. 51%	[131]
ANXA3	Western blot	AUC = 0.687	[143]
CD105	ELISA	AUC = 0.72	[144]
CD90/Thy-1	ICAT labeling, LC-MS/MS	Elevated in urine of PCa patients	[145]
CD14	MALDI-TOF, nanoLC-ESI-MS/MS	Spe. 84-100%	[146]
EN2	ELISA	Sen. 66%, Spe. 88%	[147]
ZAG	Western blot	AUC = 0.68	[148]
Fibronectin	LC-MS/MS, qRT-PCR	Sen. 75%, Spe. 50%	[149]
HP, AMBP	2-D DIGE, MALDI-MS, IPA analysis	HP, AUC = 0.723; AMBP, AUC = 0.738	[150]
UGM	N-glycosylation profiling	AUC = 0.71	[151]
B2M, PGA3, MUC3	iTRAQ, Western blot	AUC = 0.81 (3 proteins + PSA)	[5]
PSA glycoform	LC-MS	Sen. 87.5%, Spe. 60%	[152]

Abbreviations: AUC, area under the curve; Sen., sensitivity; Spe., specificity; uTF, tissue factor in urine; AMACR, α-methylacyl-CoA racemase; MCM5, minichromosome maintenance 5 protein; TB-15, thymosin β-15; MMP, matrix metalloproteinases; ANXA3, annexin A3; CD105, endoglin; CD90/Thy-1, cluster of differentiation 90; CD14, cluster of differentiation 14; EN2, engrailed-2; ZAG, zinc α2-glygoprotein; HP, haptoglobin; AMBP, α-1-microglobulin/bikunin precursor; UGM, urinary glycoprofile marker; β2M, β-2-microglobulin; PGA3, pepsinogen 3, group 1; MUC3, intestinal mucin 3; PSA, prostate specific antigen.

## CONCLUSIONS

Urine is an ideal body fluid and liquid biopsy resource for the identification and measurement of protein biomarkers [24] for prostate cancer, permitting easy, non-invasive sample collection. Urine based biomarkers offer the potential for home testing which would facilitate diagnosis and monitoring of prostate cancer patients. In addition, urinary protein biomarkers might also reflect phenotypic cellular changes of malignancy and complement or surpass biomarkers [135] from other biofluids or tissues. Genomic studies could also be enhanced by urinary protein biomarker findings thus improving the management of prostate cancer including earlier diagnosis and selection of best treatment, ultimately leading to improved outcomes [24]. Novel and accurate biomarkers could also help to identify patients who can benefit from follow-up on an active surveillance program for low-risk PCa [136]. However, current development of urinary protein biomarkers for PCa is facing numerous limitations and challenges. Most of the published studies are retrospective, use different biopsy and urine collection protocols, vary in study design and population characteristics and utilize arbitrary definitions of clinical significance and disease progression. There is also limited information regarding to intermediate endpoints and missing data with respect to long-term readouts such as time to metastasis or prostate cancer-specific mortality [136].
